# Attractive “Quiet” Courtyards: A Potential Modifier of Urban Residents’ Responses to Road Traffic Noise?

**DOI:** 10.3390/ijerph7093359

**Published:** 2010-08-30

**Authors:** Anita Gidlöf-Gunnarsson, Evy Öhrström

**Affiliations:** The Sahlgrenska Academy at the University of Gothenburg, Occupational and Environmental Medicine, Box 414, SE-405 30 Gothenburg, Sweden; E-Mail: evy.ohrstrom@amm.gu.se (E.Ö)

**Keywords:** road-traffic noise, “quiet” side, “quiet” courtyard, annoyance, perceived soundscape, restorative environments

## Abstract

The present paper explores the influence of the physical environmental qualities of “quiet”. courtyards (degree of naturalness and utilization) on residents’ noise responses. A questionnaire study was conducted in urban residential areas with road-traffic noise exposure between *L*_Aeq,24h_ 58 to 68 dB at the most exposed façade. The dwellings had “quiet” indoor section/s and faced a “quiet” outdoor courtyard (*L*_Aeq,24h_ < 48 dB façade reflex included). Data were collected from 385 residents and four groups were formed based on sound-level categories (58–62 and 63–68 dB) and classification of the “quiet” courtyards into groups with low and high physical environmental quality. At both sound-level categories, the results indicate that access to high-quality “quiet” courtyards is associated with less noise annoyance and noise-disturbed outdoor activities among the residents. Compared to low-quality “quiet” courtyards, high-quality courtyards can function as an attractive restorative environment providing residents with a positive soundscape, opportunities for rest, relaxation and play as well as social relations that potentially reduce the adverse effects of noise. However, access to quietness and a high-quality courtyard can only compensate partly for high sound levels at façades facing the streets, thus, 16% and 29% were still noise annoyed at 58–62 and 63–68 dB, respectively. Implications of the “quiet”-side concept are discussed.

## 1. Introduction

For most people, the home is a place for rest and relaxation and where relief from stress and demands of everyday life is sought. It is therefore essential that the housing environment be designed to support restoration, e.g., [1,2]. However, many residents, particularly in urban areas, are exposed to high levels of road traffic noise in and around their homes. These noise levels far exceed what characterizes a healthy and sustainable environment and may cause adverse health effects, such as sleep disturbances, general annoyance, speech interference, stress-related symptoms, and increased risk for hypertension and cardio-vascular disease, e.g., [3–9]. To improve the residential environment, current traffic noise abatement measures typically focuses on limiting noise exposure on the noisiest side of the buildings through noise barriers, improvements of window and façade insulation, and also recently by applying low-noise road surface, although these measure’s efficiency to reduce adverse noise health effects seldom are evaluated (for rare cases see e.g., [10–12]).

Recently, the “quiet”-side concept has been investigated within the research program “Soundscape Support to Health” as a tool for improving sound environments in residential areas [13,14]. One approach to create such “quiet” sides and “quiet” courtyards is to erect shielding buildings that fill existing gaps through which traffic noise penetrates. Results from the research program show that adverse health effects from road traffic noise (e.g., noise annoyance, sleep disturbances) are reduced when one or several rooms in dwellings face a “quiet” side with low sound levels from road traffic (*L*_Aeq,24h_ < 48 dB façade reflex included) and other sound sources (e.g., ventilation units), [15]. Increased perceived control of the noise exposure and opportunities to reduce the amount of time the individual is exposed to stress from noise are factors suggested for explaining this modifying effect. Although the results indicate that access to quietness is a key factor for altering resident’s noise responses, it remains unclear if these responses also are influenced by how the “quiet” courtyards are designed with respect to various physical environmental aspects. The present paper explores this issue.

There has been a growing recognition in the noise annoyance literature that built environment conditions are associated with people’s responses to traffic noise, e.g., [16,17]. Previous field studies have shown that the aesthetic/natural appearance of an exposed site moderates noise annoyance. In a large survey on nearly 3,000 people in 53 residential sites of the Greater London Council, Langdon [18] found that high neighbourhood quality (aesthetic appearance, presence of greenery) lowered dissatisfaction with traffic noise to a significant degree. Kastka and Noack [19] concluded from their research that aesthetically attractive streets, which gave an impression of having an =atmosphere of cosiness’ and =offering relief to the mind’ decreased road traffic noise annoyance compared to non-attractive streets (p. 358). Results from an explorative field study by Fyhri and Klæboe [20] show that those who visually judged the nearest blocks of their street as beautiful (e.g., presence of nature/vegetation, perceived maintenance of the buildings) also were less annoyed by road traffic noise. These field studies primarily deal with how the visual impression of the traffic exposed roads or the nearest surroundings influences noise annoyance and the interaction between the visual environment and the sound environment. However, little attention has been given to aspects in the residential area and nearby surroundings that go beyond the purely visual influence on noise responses. For example, green areas/parks nearby one’s dwelling are not only visually attractive, they may also give opportunities for escaping noise and the stressful rhythm of the city and be a place for outdoor activities that support restoration, which may moderate noise responses [21].

With regard to “quiet” courtyards, which are the focus in the present study, they provide not only a certain visual aesthetic view from the home depending on how the courtyards are designed. They also give access to a space with low sound levels from road traffic, and in addition and perhaps more importantly, the courtyards can be experienced as a useful or an unusable area. A courtyard that supports or not supports resident’s needs for doing various outdoor activities, such as to rest/relax, to read a book, to sunbathe, to have contact with neighbors, and be a place for children to play may influence overall residential satisfaction and responses to noise exposure.

In the literature, the courtyard concept is not clearly defined. It is often used to describe the space between residential buildings. Larsvall [22] defines the courtyard as “the non-built parts that are established by surrounding buildings and enclosures”. Thus, it is linked to the building/s and belongs to the residents. Many aspects characterize a courtyard and can shape the perception and satisfaction of it, e.g., number of residents, accessibility, social relations among the residents, safety perception, *etc.* [23]. The most highly valuable physical environmental aspects mentioned in the literature are presence of natural elements (e.g., trees, green surface, flowers, water, sufficient light and shadow, garden plots), protection from disturbing noise, shelter from cold winds, play areas for children, and places to sit and relax e.g., [23,24]. Visual and usability aspects are very much interconnected and may shape the overall residential satisfaction and responses to noise. Thus, presence of vegetation can contribute to an aesthetically pleasing environment and can also directly influence outdoor use [25], which increase chances for neighbors to interact with each other and to experience attachment and satisfaction with the residential area [26,27].

The main purpose of the present study is to explore how the physical environmental quality of “quiet” courtyards in noise-exposed residential urban areas affects responses to road traffic noise. We hypothesize that an attractive “quiet” courtyard with high physical environmental quality, over and above the effect of “quietness” *per se* will: (i) reduce resident’s noise annoyance and noise disturbed outdoor activities; and (ii) contribute to a positively perceived outdoor soundscape. To address this, “quiet” courtyards were identified and an objective courtyard-documentation was conducted on site, which included photographing and registration of various physical environmental aspects. This data was then linked to each resident and analyzed in relation to questionnaire data.

## 2. Method

### 2.1. Study Design

The *restricted* dataset utilized in the present study is based on questionnaire data obtained from a cross-sectional field study within the large multi-disciplinary “Soundscape Support to Health”-program. One of the main goals of this program is to develop methods and models for predicting and optimizing acoustic soundscapes in traffic noise exposed residential areas, with respect to desired perceived soundscapes and effects on health and well-being (e.g., annoyance, disturbed sleep) [14].

The cross-sectional field study was conducted in four city residential areas in Stockholm and Gothenburg, Sweden. Considerable effort was undertaken in designing the field study and in selecting the different study sites, the latter was based on a number of criteria related to: (a) half of the dwellings in the study areas should have similar sound levels at the most exposed side, but about 10–20 dB lower sound levels at the quieter side (*i.e.*, *L*_Aeq,24h_ = 38–48 dB façade reflex included) and the other half of the dwellings should have similar sound levels at the most exposed side, but with no access to a quieter side of the dwelling; (b) similarity in noise exposures (e.g., all dwellings in each noise category should have the same sound exposure from road traffic within a range of ±2 dB); (c) the study sites should be exposed to varying road traffic (e.g., not only light traffic or only heavy vehicles); (d) no other dominating noise sources should be present (e.g., rail-or aircraft noise and noise from ventilation in the courtyards); (e) similar houses according to height and type (block of flats only); (f) each dwelling should have at least two rooms in addition to a kitchen; (g) each dwelling should have access to a balcony or an outdoor space; (h) type of window should preferably be known; (i) the population’s age and number of people born in other countries should not vary to a great extent; and (j) if possible only people who had resided in the dwelling for at least one year should be selected to participate.

For selection of dwellings and calculations of individual noise dose immission levels, study sites and buildings were examined by aerial photographs and documented in 1:4,000 scale map format with elevation contours. Plan drawings of dwellings and data from the questionnaire of stated floor level and the location of the balcony, bedroom windows, and living room windows were also used. For more details, see also [15].

### 2.2. Noise Exposure

To link sound exposure and adverse health effects, we determined outdoor sound levels at both sides of *each* dwelling by: (1) long-term measurements (at least one complete week) in both directly exposed and shielded areas (not during holidays or other times when traffic might have been abnormal); (2) short-term measurements (for at least 30 min or 500 vehicles); and (3) counting of traffic data (number of light and heavy vehicles, percentage of heavy vehicles). When sufficiently exact traffic data were available from authorities, these data were used; and (4) calculations of road traffic noise levels (*L*_Aeq,24h_, *L*_night,22-06_, and *L*_AFmax_) for each dwelling based on traffic input and geometrical data for the site. For more details of sound exposure assessments, see [15,28,29]. The sound level values at the “quiet” side include the façade reflex according to the new calculation model developed in the research program [28,29]. The particular storey was considered in the calculations.

### 2.3. Participants and Noise Exposure Categories

The *restricted* dataset utilized in the present study (*n* = 385) is based on questionnaire data obtained from 956 residents exposed to sound levels between *L*_Aeq,24h_ 45 to 68 dB. One individual between 18 and 75 years of age in 1,625 households were originally selected (59% response rate). If there were two or more persons in the household, the one who had his/her birthday closest to the date of the first distribution of the questionnaire was chosen. The responses revealed that 458 individuals had access to a “quiet” side of their dwelling while 498 had no such access. All residents in the *restricted* dataset had access to a “quiet” side and were exposed to sound levels between *L*_Aeq,24h_ 58 to 68 dB at the most exposed facade. The remaining residents with no access to a “quiet” side and with sound levels below 58 dB were excluded (the latter because very few individuals had sound levels between 48 to 57 dB and those exposed to sound levels between 45–46 dB represented a reference site having a good sound environment). We formed two sound level categories: 58–62 dB (*n* = 241) and 63–68 dB (*n* = 144).

### 2.4. The Physical Environmental Quality of Courtyards

All buildings in the study sites were blocks of flats and 90% of them were 3–5 stories in height. The buildings had various types of more or less closed courtyards in relation to the trafficked street (see Table 1). A majority (57%) of the buildings consisted of a single building, one tenth was linked to another building, 14% were linked with two other buildings (half-closed courtyard) and the fourth category of buildings had a closed courtyard (19%).

The mean sound levels (3 m from the façade) at the courtyard side did not differ largely depending on courtyard type: open (48.2, SD = 1.76), half-open (48.1, SD = 0.68), half-closed (49.5, SD = 1.20), and closed (49.8, SD = 0.41). Each of the “quiet” courtyards and all façades were documented with photos by one project assistant and the same assistant also conducted a detailed registration of various physical environmental aspects that typically was expected to be present in courtyards (Table 1).

The aspects were: presence of trees and bushes, presence of flowers in pots/flowerbeds, amount of green surface, amount of asphalt, presence of garden furniture, presence of playground, size of the courtyard, type of terrain, courtyard facing weather quarter, type of courtyard in relation to the trafficked street, access to laundry, presence of garbage recycling, presence of car park/garage, and presence of bicycle park. This data were linked to each resident who participated in the questionnaire study.

Based on results from previous research, e.g., [22–24,26,30–33], we decided to select a smaller set of aspects due to their documented influence on residential satisfaction and well-being and their importance in the design of residential outdoor areas. The aspects are related to “courtyard utilization” and “naturalness”. “Courtyard utilization”, *i.e.*, the potential for carrying out various activities, such as opportunities for rest and to meet people, provided by: (i) presence of outdoor furniture; and (ii) playground for children; and “Naturalness”; (iii) presence of flowers in pots/flowerbeds and (iv) the type of weather quarter the courtyard is facing (north = 0, east = 1, south = 2, west = 3; a higher scale value indicate higher quality because of better opportunity for the sun light to reach the courtyard; weather quarter west is ranked as highest since it gives the best opportunity for sunlight during the afternoon when people are home from work and school).

**Figure 1.**
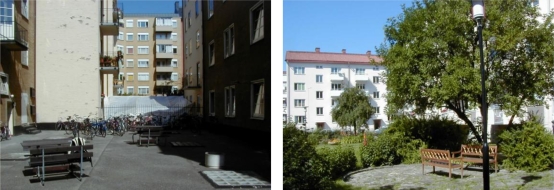
Examples of “quiet” courtyards in the study with low (left) and high environmental physical quality (right).

We wanted to include the amount of green surface as well in the analysis since we thought it was an important natural courtyard aspect. However, given its unevenly distribution and that all residents had either some green surface in the courtyard, or trees/bushes, or both of these aspects, we considered that some type of greenery was present in all courtyards and, therefore, we decided to exclude green surface in the assessment of courtyard quality. Since the amount of green surface and asphalt assessed the same thing, the asphalt aspect was also excluded. For the first three aspects (i–iii), a value of 1 was given as a positive response if the aspect was present. Adding the responses of the four aspects forms a score that represents the physical environmental quality of each courtyard (possible range 0–6). Based on the median value (Md = 3, range 1–6), we formed two groups: residents with a score ≤3 were classified as having access to a “quiet” courtyard with low physical environmental quality (*n* = 239) and residents with a score ≥4 were classified as having access to a “quiet” courtyard with high physical environmental quality (*n* = 146) (Figure 1).

Table 2 shows the distribution of residents in two courtyard quality-groups (low and high) and two sound level categories (*L*_Aeq,24h_ = 58–62 and 63–68 dB), as well as means and standard deviations of the courtyard’s physical environmental quality scores (within parentheses).

### 2.5. Questionnaire

The design of the questionnaire is based on previous research on adverse health effects of noise [5,34] and on methods for assessing soundscapes [35,36]. The questionnaires were sent by post together with an introductory letter that presented the study as a study on well-being and the general living environment. One or two reminder letters were sent to those who didn’t answer within 10 days. In the present study, five main sets of data were utilized from the questionnaire (for detailed descriptions of the questionnaire, please consult [15]). The five sets were: (1) person factors (gender, age, occupation, longstanding illness and self-estimated noise sensitivity; (2) general noise annoyance; (3) disturbances of outdoor activities due to road traffic noise; (4) perceived soundscape; and (5) satisfaction with the residential area.

1. *Person factors*. The residents were to report on gender, age, and occupation. Longstanding illness was assessed as frequencies of “yes/no” responses. Sensitivity to noise was assessed by asking the respondents the following: “How would you in general describe your sensitivity to noise?” A 4-point category scale was used ranging from “not at all sensitive” = 1 to “very sensitive” = 4.
2. *General noise annoyance* was assessed with an internationally adopted and standardized annoyance scale for comparison with internationally executed studies on annoyance [37]. The scale assessed road traffic noise annoyance at home (last 12 months) on a 5-point category scale (“not at all” = 1, “slightly” = 2, “moderately” = 3, “very” = 4, and “extremely” = 5).
3. *Disturbances of outdoor activities due to road traffic noise* were assessed by a set of items referring to relaxation, communication, and staying outdoors. Each item was evaluated from two questions on how often (“never” = 0, “sometimes” = 1, and “often” = 2) and to what degree (“not very disturbing” = 2, “rather disturbing” = 3 and “very disturbing” = 4) the activities were disturbed by road traffic noise. A disturbance score ranging from 0 to 6 was constructed, where the value on frequency was added to the value on degree of disturbance.
4. *Perceived soundscape*. Identification of sound sources is valuable when describing residential sound environments. Some sounds are perceived as intruding and some sounds are perceived as they belong in the residential sound environment [35]. Certain “natural sounds” (e.g., bird song, water) are commonly perceived as pleasant [36,38]. The residents were asked to report on how often (during the last 12 months) they usually heard 14 various sound sources (private cars, trucks, buses, motorcycles, aircraft, railway, gardening machinery, ventilation, TV/radio, sound of steps, dogs/other pets, birds, children playing, and people talking) when they were outdoors close to their dwelling. For each sound source, a 4-point category scale was used ranging from “hear seldom/never” to “hear almost always”.
5. *Satisfaction with the residential area* was assessed with one question on a 5-point category scale ranging from “very good” = 1 to “very bad” = 5.

### 2.6. Statistical Analyses

Differences in proportions for categorical variables were determined with the Chi-square test (χ^2^) and differences in mean values were determined with the *t*-test. A binary logistic regression analysis was used to estimate the relationship between noise annoyance (0 = not annoyed, 1 = annoyed), the level of road traffic noise exposure (two categories; *L*_Aeq,24h_ = 58–62 and 63–68 dB), and courtyard quality (two categories; low and high). With a 2 × 2 ANOVA-analysis, the main and interaction effects of road traffic noise exposure (two categories; *L*_Aeq,24h_ = 58–62 and 63–68 dB) and courtyard quality (two categories; low and high) on noise disturbed outdoor activities (summed score of three items that formed an index) was investigated. Reliability of the index measure (internal consistency) was analyzed with Chronbach’s α. A value of *p* < 0.05 was considered statistically significant. Statistical analyses were conducted with PASW Statistics 18.

## 3. Results

### 3.1. Person Factors and Sound Levels at the Exposed and “Quiet” Sides of the Dwelling

Across the two sound exposure categories of *L*_Aeq,24h_ = 58–62 and 63–68 dB, the courtyard groups are not significantly different (*p* > 0.05) in gender, occupation, long-standing illness, or sensitivity to noise (Table 3). Residents with high courtyard quality in the 58–62 dB sound level category are significantly older than residents with low courtyard quality (mean = 47.2, SD = 13.86 *vs.* mean = 43.3, SD = 14.76 years of age, respectively) and are exposed to slightly higher sound levels at the noise exposed façade (mean = 61.1 dB, SD = 0.82 *vs.* mean = 60.4 dB, SD = 1.31 *L*_Aeq,24h_ dB for high and low courtyard quality groups, respectively). Although significant, this difference in mean sound level at the most exposed façade is exceedingly small and, therefore, considered as negligible. No significant mean sound level differences at the “quiet” side of the buildings were found.

### 3.2. High-Quality “Quiet” Courtyards are Associated with Lower General Noise Annoyance

The influence of noise levels and courtyard quality on road traffic noise annoyance was explored by a binary multiple logistic regression analysis (*n* = 385). The model contained general noise annoyance as a binary variable (0 = not at all/slightly annoyed; 1 = moderately/very/extremely annoyed), exposure to road traffic noise (*L*_Aeq,24h_ = 58–62 and 63–68 dB), and courtyard quality (0 = low, 1 = high). Age was included as a covariate. Table 4 shows that general annoyance is significantly related to noise exposure (OR 1.99; 95% CI 1.24 to 3.13) and courtyard quality (OR 0.59; 95% CI 0.36 to 0.96).

**Figure 2.**
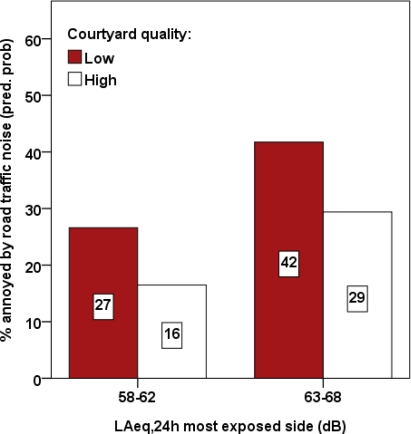
Percentage of noise annoyed residents due to road traffic when being at home in relation to courtyard quality and sound levels.

As can be seen in Figure 2, the percentage of noise annoyed residents was significantly lower across the two sound level categories among those who had high (16% and 29%) than low-quality “quiet” courtyards (27% and 42%).

### 3.3. High-Quality “Quiet” Courtyards is Associated with Less Noise-Disturbed Outdoor Activities

**Figure 3.**
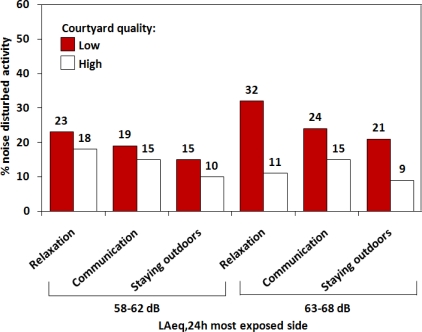
Noise disturbed outdoor activities (%) in relation to courtyard quality and sound levels.

Figure 3 presents the percentage of residents reporting noise-disturbed outdoor activities with a score above three. This includes individuals who report that they are alternatively sometimes and rather disturbed (score 4), often and rather disturbed (score 5), or often and very disturbed (score 6). Dummy variables were formed (score ≤3 = 0 and ≥4 = 1) and the differences between groups of residents with low and high courtyard quality were determined by χ^2^-tests.

As depicted in Figure 3 for both sound level categories, fewer residents reported noise-disturbed outdoor activities if they have access to a courtyard with high compared to low physical environmental quality (range 4 to 21 percentage points less). However, a significant courtyard effect is observed only in the 63–68 dB category for disturbed relaxation (32 *vs.* 11% for low and high courtyard quality, respectively; χ^2^_1_ = 7.2, *p* < 0.01).

To further determine the role of courtyard quality in the relationship between road traffic noise exposure and noise disturbed outdoor activities, a two-way ANOVA was conducted (2 sound level categories × 2 courtyard quality categories). Disturbed relaxation, communication and outdoor stay were summed to form an index of noise-disturbed outdoor activities (Chronbach’s α = 0.72).

The ANOVA yielded a significant main effect of courtyard quality [*F*(1,381) = 7.27, *p* < 0.01] on noise-disturbed outdoor activities, but noise exposure had no such significant main effect [*F*(1,381) = 0.42, *p* > 0.05], although residents with low courtyard-quality reported more activity disturbance in the 63–68 dB category. This was not the case for the group with high courtyard quality. No significant interaction effect was found between road traffic noise exposure and courtyard quality [*F*(1,381) = 0.66, *p* > 0.05].

### 3.4. High-Quality “Quiet” Courtyards Influence the Perceived Residential Soundscape

The perceived residential soundscape when being outdoors close to the dwelling differed somewhat between the two courtyard groups. In both sound level categories, the percentages of residents hearing sounds “often” or “almost always” from traffic (private cars, trucks, buses, motorcycles, aircraft, railway), garden machinery, ventilation, and TV/radio were overall similar among residents in the low and in the high courtyard quality group (differences ranged between 1 to 9 percentage points). However, larger differences between the courtyard groups were found for natural and human sounds (range 8 to 24 percentage points), as presented in Table 5.

In both sound level categories, a significantly higher percentage of residents (*p* <0.05) heard bird song frequently when being outdoors if they had access to a courtyard with high physical environmental quality. In the 58–62 dB category, this was also found for hearing children playing and people talking (*p* <0.05), but the same results for the highest sound level category were not significant (Table 5). When analyzing the whole sample (not divided into two sound level categories), significant high and low courtyard-group differences were found for hearing the natural and human sound sources (bird song = 58% *vs.* 42%, χ^2^_1_ = 8.79, *p* < 0.01; children playing = 50% *vs.* 36%, χ^2^_1_ = 7.14, *p* < 0.01; people talking = 76% *vs.* 56%, χ^2^_1_ = 14.89, *p* < 0.01).

### 3.5. High-Quality “Quiet” Courtyards are Associated with Satisfaction of the Residential Area

Many of the residents were satisfied with their residential area and rated it as good or very good. In both sound level categories, there was a tendency that high courtyard quality was associated with a higher proportion of residents rating their residential area as very good (11 and 6 percentage points more in the 58–62 and 63–68 dB categories, respectively; *p* > 0.05). When analyzing the whole sample (not divided into the two sound level categories), courtyard quality significantly influenced residential satisfaction (52% *vs.* 42% in high and low courtyard categories, respectively; χ^2^_1_ = 3.85, *p* < 0.05).

## 4. Discussion

The overall results of this explorative study indicate that the physical environmental quality of “quiet” courtyards influenced residents’ responses to noise. In the logistic regression analysis, the exposure-response relationship between road traffic noise and general annoyance at home was significantly modified by courtyard quality indicated by lowered odds of falling into the annoyance group. Figure 2 suggests that access to a high-quality “quiet” courtyard lower the percentage of annoyed residents by 9 and 13 percentage points depending on the sound level from road traffic at the most exposed side of the dwelling (58–62 and 63–68 dB, respectively).

The descriptive analyses of the questions on road-traffic disturbed outdoor activities follow the same direction: fewer residents in high than low-quality courtyards reported noise-disturbed outdoor activities. Furthermore, we found a significant main effect of courtyard quality on the index of noise-disturbed outdoor activities in the ANOVA analysis, but neither a significant main effect of noise exposure nor an interaction effect between road traffic noise exposure and courtyard quality.

The above findings indicate that noise levels at the most exposed side of the dwelling have less impact on annoyance and disturbance responses under high than low courtyard quality conditions. Based on previous research on visual aesthetic factors and noise responses one plausible interpretation of the results is that high-quality courtyards have a more attractive overall visual aesthetic appearance that increases the resident’s satisfaction of their dwelling and housing environment, which may have lead to a modification of their annoyance reactions [18–20,39]. Research on restorative environments suggest that visual aesthetic scenes containing natural elements have the ability to restore depletion of attentional capacity caused by stimuli overload (from e.g., chronic noise exposure) and directed attention fatigue [26,40,41]. Such natural scenes are also associated with activation of mental processes and states that reduce stress and promote well-being [42,43]. Kaplan [32] found that the view from one’s home over a garden, flowers or a landscaped area strongly increased resident’s neighborhood satisfaction and well-being. She suggests that this kind of home view has the ability to provide residents with ‘micro-restorative experiences’ (p. 538). Thus, a “quiet” and visually attractive courtyard with natural elements may assist in shifting noise-exposed resident’s attention from effortful (e.g., focus on traffic noise) to effortless (e.g., experiences of tranquility, positive feelings).

However, apart from the “pure” visual influence that may modify noise responses, possibilities to use the courtyard for various outdoor activities are probably also of importance in this context. One significant courtyard aspect related to this is how much the courtyard is reached by the sun [24,44]. Of the low-quality courtyards, 46% faced north, but none of the high-quality courtyards did so. In Sweden, sunshine is highly valued and often necessary for comfortable temperatures. The Swedish National Board of Housing, Building and Planning recommend that green spaces and outdoor plots in connection with residential buildings have at least 5 hours of sunshine at vernal- and autumnal equinox, in [22]. Spaces sufficiently reached by the sun may also be more cared for and can better provide conditions for growing flowers and other plants, thereby increasing attractiveness (94% and 47% of the high and low-quality courtyards had flowers, respectively). Flowers and greenery may also attract visits of birds that can contribute to a positively perceived soundscape, e.g., [36,38,45,46]. In the whole sample, bird song was heard by a significantly higher number of residents with high than low-quality courtyards. This was also true for sounds from children playing and people talking, which may indicate that high-quality courtyards are used more often by children and adults.

It can be argued that human sounds, specifically from children playing, can be perceived as unpleasant, particularly among elderly people. However, for both sound level categories we found no indications that older (>45 years of age) residents were significantly more disturbed by sounds from children indoors and outdoors (11-point scale ranging from “not at all disturbed” to “extremely disturbed”) than the younger (<45 years of age) residents (58–62 dB, mean disturbance = 1.00 *vs.* 0.83, *t*_234_ = 0.79, *p* > 0.05; 63–68 dB, mean disturbance = 0.69 *vs.* 1.05, *t*_139_ = −1.17, *p* > 0.05).

Presence of play ground and outdoor furniture/benches support outdoor stay and are important for affording social functions [23,25,33]. Skjaeveland and Garling [27] found that neighborhoods characterized by the presence of structuring elements (e.g., fences, planting, and furniture) influenced patterns of sociospatial relationships. Gehl [31] explored activities in residential streets with light traffic and found that streets with so called “soft edges” (e.g., front yards) increased long duration activities provided there were certain physical arrangements (benches, opportunities for children to play). He concluded that (p. 97) “when the physical layout is too poor, the majority of people simply do not leave their home”. Our findings are in line with this reasoning – high-quality “quiet” courtyards can encourage and support residents’ outdoor stay by providing a place for children to play and adults to get together, or to do other outdoor activities.

### 4.1. Limitations

As part of the research program “Soundscape Support to Health”, this study was originally designed for investigating resident’s responses to noise in relation to sound levels and access to a “quiet” side of one’s dwelling, and the selection of the residential areas was based on this and other criteria (e.g., similarity in road-traffic sources, population characteristics *etc*.). Therefore, the variation of courtyard quality with respect to the presence and amount of the different physical environmental aspects, as well as its assessment was affected. However, the consistent associations between the environmental quality of the “quiet” courtyards and the resident’s responses, as well as no significant influences of demographic and person factors (except for age in the 58–62 dB category) indicate that the present study sample and the constructed courtyard quality scale was relevant. Thus, the physical courtyard aspects that were chosen in the present study have also in other investigations shown to be important for residents’ satisfaction with their residential situation and their perception of the urban environmental quality, e.g., [22,27,31,39]. Similar aspects are also commonly mentioned in judgments of residential settings and of courtyard quality. For example, in reports from the Swedish National Board of Housing, Building and Planning, in [22], the usability of courtyards is evaluated against presence of greenery, trees and bushes, playground, amount of sun hours, *etc*. Although the results in this cross-sectional study are promising, further research ought to investigate the generalizability of the present findings.

## 5. Concluding Remarks

It is clear that the “quiet”-side concept is beneficial for people’s health and well-being in a long-term perspective and for developing a sustainable built environment. We believe that the acoustic soundscape in many cases can easily be changed with rather low costs so that the access to quietness increases, exposure to traffic noise decreases and, thereby, the adverse effects of traffic noise are reduced. In existing residential settings, an active way to increase the access to quietness is to erect shielding buildings that fill existing gaps through which traffic noise penetrates and spoils the shielding effects. In many city areas, closed and rather “quiet” courtyards or other building structures with good shielding effects already exist that create noise-protected areas. It is, however, common in cities that housings contain small dwellings with no direct access to the “quiet” side. Furthermore, older houses are more often planned in such a way that kitchens rather than bedrooms and living rooms are facing the courtyards. This must be taken into account in city renewal projects. Still, only quietness is not a sufficient criterion for a good built environment. Our results indicate that it is also important to create attractive high-quality “quiet” courtyards and other shielded spaces that can offer urban residents a positively perceived sound environment, an attractive visual appearance, and opportunities for rest, play, social contact, and to do other activities.

To fulfill the long-term goal for a healthy built environment, it is desirable to improve existing sound environments and to avoid new residential buildings in heavily noise exposed areas. The implementation of the “quiet”-side concept [13] have proved to be of great importance for reducing the adverse effects of road traffic noise and for promoting sound environments that support health and well-being. However, although results from the “Soundscape Support to Health”-program show that the difference in adverse health effects is substantial between those who have and those who do not have access to a “quiet” side [15] and that courtyard quality is of importance, access to quietness and high-quality courtyards only compensates partly for high noise levels at façades facing the streets (16% and 29% were still noise annoyed at 58–62 and 63–68 dB, respectively). This is important to realize since there is a risk that the “quiet”-side concept is misused as an argument for planning and building new residential areas in heavily traffic-noise exposed environments. The present results and findings in the program [15] clearly show that there is a noise limit (about *L*_Aeq, 24h_ = 60 dB from road traffic at the exposed façade) whereby the “quiet” side loses its strong beneficial effect and this is necessary to take into account in future development plans for traffic and housing.

## Figures and Tables

**Table 1.** Distribution in percent for registered physical environmental aspects of “quiet” courtyards.

**Courtyard aspects**	**Registration category (%)**	
Trees and bushes	Yes = 97	No = 3
Flowers in pots/flowerbeds	Yes = 65	No = 35
Green surface	≤30% = 24; 40–65% = 14; ≥70% = 62	
Asphalt	≤30% = 62; 40–65% = 14; ≥70% = 24	
Benches/garden furniture	Yes = 74	No = 26
Playground	Yes = 35	No = 65
Size of the courtyard	Small = 19; Medium 42; Large =39	
Terrain	Hilly = 57; Flat = 43	
Courtyard facing weather quarter	North = 29; East =38; South = 31; West = 3	
Type of courtyard in relation to the trafficked street: *		
One building—open	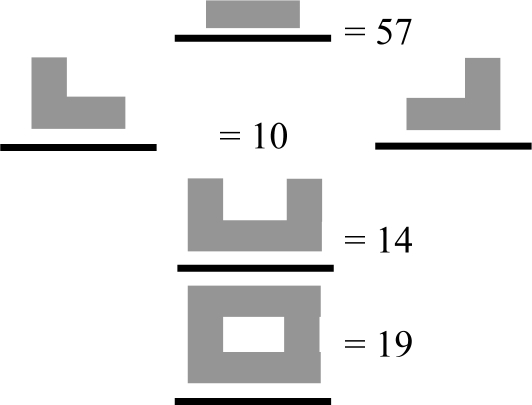	
Two buildings linked to each other—half-open		
Three buildings linked to each other—half-closed		
Four buildings linked to each other—closed		
Laundry	Yes = 100	No =0
Garbage recycling	Yes =19	No = 81
Car park/garage	Yes =4	No = 96
Bicycle park	Yes =75	No = 25

* The black lines represent the trafficked streets and the grey blocks represent the buildings.

**Table 2.** Distribution of residents (frequency) in two courtyard quality groups and two sound level categories.

	Courtyard quality groups*		

**Sound levels at the most exposed side (*L*_Aeq,24h_ dB)**	**Low**	**High**	**Total number of residents**
58–62 dB	141 (1.6, 0.77)	100 (4.5, 0.67)	241
63–68 dB	98 (2.1, 0.77)	46 (4.4, 0.50)	144
Total number of residents	239	146	385

* Values within parentheses show means and standard deviations for courtyard quality scores.

**Table 3.** Questionnaire responses on background variables and measured *L*_Aeq,24h_ dB in relation to sound levels and access to a courtyard with low and high physical environmental quality.

	
	**58–62 dB^a^**			**63–68 dB^a^**		

**Variables**	**Low^b^ (*n* = 141)**	**High^b^ (*n* = 100)**	***p*^c^**	**Low^b^ (*n* = 98)**	**High^b^ (*n* = 46)**	***p*^c^**
Gender: (%)			n.s			n.s
Female	61	53		50	50	
Male	39	47		50	50	
Age: Mean (SD)	43.3(14.76)	47.2(13.86)	0.04	42.7(17.24)	43.5(14.61)	n.s
Occupation: (%)			n.s			n.s
Employed	72	69		62	63	
Studying	11	10		14	15	
Unemployed	2	4		2	9	
Retired	12	15		20	11	
Working in the home/Other	3	2		2	2	
Longstanding illness: (% yes)	30	33	n.s	34	30	n.s
Sensitive to noise: Mean (SD)	2.3(0.82)	2.2(0.81)	n.s	2.2(0.90)	2.4(0.76)	n.s
*L*_Aeq, 24h dB_: Mean (SD)						
Noise-exposed side	60.4(1.31)	61.1(0.82)	<0.001	64.2(1.49)	63.8(0.70)	n.s
“Quiet” side	48.6(1.89)	48.9(1.69)	n.s	48.5(1.00)	48.8(1.31)	n.s

a Sound levels (*L*_Aeq,24h dB_) at the most exposed side of the dwelling;

b Physical environmental quality of the courtyard;

c Differences between groups of residents with low and high physical environmental quality of their courtyard were determined by χ^2^-tests of percentages and by *t*-tests of mean values.

**Table 4.** Results of multiple logistic regression analysis with 95% confidence intervals predicting noise annoyance from noise exposure and courtyard quality.

**Variables**	***b***	***p*-value**	**OR^a^**	**95% CI**
Noise exposure (*L*_Aeq,24h_dB)	0.68	0.004	1.99	1.24–3.13
Courtyard quality	−0.53	0.035	0.59	0.36–0.96

a Adjusted for age.

**Table 5.** Identification of sound sources (%) in relation to courtyard quality and sound levels.

	
**Sound sources**	**58–62 dB**			**63–68 dB**		

	**Courtyard quality**			**Courtyard quality**		

	**Low**	**High**	***p*^a^**	**Low**	**High**	***p*^a^**
Bird song	39	54	0.02	46	65	0.03
Children playing	41	56	0.02	29	37	0.33
People talking	58	82	0.00	54	63	0.29

a Dummy variables were formed (hear seldom, never/hear sometimes = 0; hear often/hear almost always = 1) and the differences between groups of residents with low and high physical environmental quality of their courtyard were determined by χ^2^-tests of percentages.
